# Factors associated with self-reported health: implications for screening level community-based health and environmental studies

**DOI:** 10.1186/s12889-016-3321-5

**Published:** 2016-07-26

**Authors:** Jane E. Gallagher, Adrien A. Wilkie, Alissa Cordner, Edward E. Hudgens, Andrew J. Ghio, Rebecca J. Birch, Timothy J. Wade

**Affiliations:** 1 Environmental Public Health Division, National Health and Environmental Effects Research Laboratory, U.S. Environmental Protection Agency, Mail Drop: 58C, Research Triangle Park, NC 27711 USA; 2 Oak Ridge Institute for Science and Education, Environmental Public Health Division, National Health and Environmental Effects Research Laboratory, U.S. Environmental Protection Agency, Mail Drop: 58C, Research Triangle Park, NC 27711 USA; 3 Sociology Department, Whitman College, 345 Boyer Ave, Walla Walla, WA 99362 USA; 4 Westat, 1600 Research Blvd, Rockville, MD 20850 USA

**Keywords:** Self-reported health, Screening level health assessment, Clinical measures, Metal mixtures analyses, NHANES

## Abstract

**Background:**

Advocates for environmental justice, local, state, and national public health officials, exposure scientists, need broad-based health indices to identify vulnerable communities. Longitudinal studies show that perception of current health status predicts subsequent mortality, suggesting that self-reported health (SRH) may be useful in screening-level community assessments. This paper evaluates whether SRH is an appropriate surrogate indicator of health status by evaluating relationships between SRH and sociodemographic, lifestyle, and health care factors as well as serological indicators of nutrition, health risk, and environmental exposures.

**Methods:**

Data were combined from the 2003–2006 National Health and Nutrition Examination Surveys for 1372 nonsmoking 20–50 year olds. Ordinal and binary logistic regression was used to estimate odds ratios and 95 % confidence intervals of reporting poorer health based on measures of nutrition, health condition, environmental contaminants, and sociodemographic, health care, and lifestyle factors.

**Results:**

Poorer SRH was associated with several serological measures of nutrition, health condition, and biomarkers of toluene, cadmium, lead, and mercury exposure. Race/ethnicity, income, education, access to health care, food security, exercise, poor mental and physical health, prescription drug use, and multiple health outcome measures (e.g., diabetes, thyroid problems, asthma) were also associated with poorer SRH.

**Conclusion:**

Based on the many significant associations between SRH and serological assays of health risk, sociodemographic measures, health care access and utilization, and lifestyle factors, SRH appears to be a useful health indicator with potential relevance for screening level community-based health and environmental studies.

**Electronic supplementary material:**

The online version of this article (doi:10.1186/s12889-016-3321-5) contains supplementary material, which is available to authorized users.

## Background

Health outcomes are multi-determined and result from complex interactions of social, cultural, economic, psychosocial, environmental, and community factors. However, this wide range of factors are typically studied in a ‘siloed’ manner [1]. Effective public health policies can be generated only if a range of risks along the complex causal chain leading to health outcomes is assessed, defined, and studied comprehensively [2].

Self-reported health (SRH) is a qualitative single-question assessment of health [3]. SRH is commonly acquired in health surveys in the United States (e.g., MacArthur Field Study of Successful Aging, Hawaii Health Survey, San Luis Valley Diabetes Study, National Risk Survey, National Health and Nutrition Examination Survey [NHANES], and Robert Wood Johnson Foundation) [4–7] and internationally (e.g., Spanish National Disability Survey,

European Organization for Research and Treatment of Cancer Quality of Life Questionnaire, National Population Health Survey, and Manitoba Longitudinal Study on Aging) [8–10]. SRH is also commonly used in psychological research, clinical settings, and in general population surveys [11].

Studies have shown that SRH is associated with lifestyle related diseases (e.g., diabetes and hypertension [12]), lifestyle habits (e.g., smoking status [13], regular physical exercise [14], obesity [15], and, most notably, subsequent mortality [4, 10]). The validity and value of SRH, with respect to mortality, is independent of clinical or physician assessments, and SRH surpasses these measures in predictive power [11]. Few studies link SRH with diagnostic clinical indicators of disease [12, 16, 17] and even fewer evaluate SRH in relation to blood or urinary based biomarkers of environmental exposure [17, 18].

Many diseases and health conditions are often not reported, thus county, state, and national surveys often have limited health outcome data [19]. Local, state, and national public health officials, exposure scientists, and environmental justice advocates would benefit from a screening level health status indicator, such as SRH, to identify potentially vulnerable communities and modifiable health risk factors. Such an indicator would also add value to studies where both environmental exposures and social determinants of health are simultaneously assessed [20].

This study investigates the utility of SRH as a general proxy for health status by investigating whether, and to what extent, SRH is associated with race/ethnicity and broad range of health-risk indicators (*N* = 57) thought to be important determinates of health. Data were extracted from NHANES and include race/ethnicity and health risk factors across six domains: sociodemographic, health care, health status (e.g., diseases/health conditions), lifestyle factors, serological clinical and nutritional indicators, and blood biomarkers of exposures for metals and volatile organic compounds.

## Methods

Physical, medical, laboratory, and respondent data from questionnaires and clinical analysis were extracted from publically available NHANES data from survey years 2003–2004 and 2005–2006. The data and more information about data collection are available online [21]. Data on SRH and a broad array of subjective and objective respondent characteristics, including sociodemographic indicators, health care, lifestyle factors, and diseases, were obtained from interviewer administrated computer-assisted personal interviews conducted at the household interview and mobile examination center [22, 23]. While all NHANES participants complete a computer assisted personal interview, full serum analysis, including chemical exposure assessment, is conducted for only a randomly selected subset of NHANES participants.

### Study population

Of the full NHANES study sample of 5214 participants between the ages of twenty and fifty, the study population for this analysis is composed of 1372 twenty to fifty year old nonsmokers with complete data on SRH and serum biomarkers. Respondents (*N* = 1731) were omitted from the analysis if their serum cotinine concentration was greater than 10 ng/mL (*N* = 1648), or if serum cotinine was missing and they self-identified as a current smoker (*N* = 81), or if both were missing (*N* = 2). We restricted the analysis to current nonsmokers due to the adverse health impact associated with smoking. We did not want to overly influence (weaken or strengthen) any potential associations between SRH and the various factors by including smokers. We verified the suspected strong relationship between SRH and smoking in preliminary analyses (not shown) that found smokers, both self-identified current smokers and participants with cotinine measurements >10 ng/mL, were twice as likely to report poor/fair health as compared to nonsmokers. An additional 2111 respondents were excluded due to missing values for benzene and/or toluene (*N* = 1948) or due to missing data for SRH and pertinent demographic, body measurement, and clinical data (*N* = 163). If data were missing from less than 20 participants for other variables, those participants were excluded from analysis using that variable. If data were missing from more than 20 participants, a “missing” category was included for analysis of that variable. Sample sizes are provided in Tables 1 and 2. Some variables of interest were only analyzed for females (e.g., ferritin, transferrin receptor, transferrin saturation, iron, hemoglobin, and total iron binding capacity); thus, the sample size for analyses that include these variables is lower.

**Table 1:** Sociodemographic characteristics of the study population (*N* = 1372 twenty to fifty year old nonsmokers)

	*N*	Weighted percent^a^ (SE)		*N*	Weighted percent^a^ (SE)
Sex			Marital Status		
Male	547	46.4 (1.4)	Married	813	63.8 (2.0)
Female	825	53.6 (1.4)	Never married	315	20.1 (1.7)
Race/Ethnicity			Other	244	16.1 (1.3)
Mexican American	350	10.8 (1.2)	Education		
Other hispanic	61	4.9 (0.9)	<9th grade	116	4.0 (0.6)
Non-Hispanic white	611	68.0 (2.5)	9–12th grade	178	8.2 (0.9)
Non-Hispanic black	285	10.9 (1.5)	High School Grad/GED	253	19.2 (1.8)
Other Race (including multi-racial)	65	5.3 (1.1)	Some College or AA degree	447	33.8 (1.8)
Country of birth			≥College Graduate	378	34.8 (2.2)
United States	969	80.4 (2.1)	Annual Family Income		
Mexico	236	7.1 (0.7)	<$20,000	288	14.1 (1.1)
Elsewhere	167	12.5 (2.0)	$20,000 to $44,999	434	26.5 (2.0)
US citizen		0.0 (0.0)	$45,000 to $74,999	268	21.6 (1.6)
Yes	1082	87.5 (1.4)	≥$75,000	382	37.8 (2.6)
No	290	12.5 (1.4)			

*Abbreviations: GED* general education development, *AA* associate degree

^a^The weighted percent adjusts for differential probabilities of selection, nonresponse, and differences between the final sample and the total population

**Table 2:** Lifestyle and health characteristics of the study population (*N* = 1372 twenty to fifty year old nonsmokers)

	*N*	Weighted percent^f^ (SE)		*N*	Weighted percent^f^ (SE)
Home ownership			Self-reported health status		
Owned or being bought	807	68.1 (2.5)	Excellent	309	24.0 (1.5)
Rented	531	30.1 (2.4)	Very good	442	35.9 (1.4)
Other arrangement	34	1.8 (0.4)	Good	434	30.9 (1.7)
Watch TV 3+ hours/day			Fair	166	8.1 (0.8)
No	948	72.5 (1.4)	Poor	21	1.2 (0.3)
Yes	424	27.5 (1.4)	High blood pressure^d^		
Worried would run out of food^a^			No	1238	88.8 (1.1)
Often true	88	4.4 (0.8)	Yes	134	11.2 (1.1)
Sometimes true	198	9.1 (1.0)	Diabetes^e^		
Never true	845	62.4 (2.4)	No	1306	95.3 (0.5)
Screened out	219	22.5 (1.6)	Yes	64	4.5 (0.5)
Missing	22	1.6 (0.4)	Missing	2	<0.3^c^
Worried couldn’t afford balanced meals^a^			Ever had asthma^e^		
Often true	44	2.6 (0.5)	No	1203	86.6 (1.0)
Sometimes true	146	6.4 (0.9)	Yes	169	13.4 (1.0)
Never true	939	66.8 (2.1)	Current asthma^e^		
Screened out	219	22.5 (1.6)	No	1268	92.1 (0.9)
Don’t know/ Missing	24	1.7 (0.4)	Yes	102	7.8 (0.9)
Health insurance			Missing	2	<0.3^c^
No	350	20.0 (1.7)	Asthma attack past year^e^		
Yes	1022	80.0 (1.7)	No	1319	95.7 (0.5)
Number of prescription medications^b^			Yes	51	4.1 (0.5)
0	833	54.3 (1.5)	Missing	2	<0.3^c^
1	245	19.6 (1.3)	Ever had thyroid problem^e^		
2	125	10.4 (1.0)	No	1289	92.6 (0.9)
3 to 4	121	11.3 (1.1)	Yes	82	7.3 (0.9)
>4	48	4.4 (0.8)	Missing	1	<0.3^c^
Body Mass Index			Ever had cancer/malignancy^e^		
Underweight (<18.5)	19	1.4 (0.4)	No	1348	97.3 (0.5)
Normal weight (18.5–< 25)	414	32.2 (1.5)	Yes	23	2.7 (0.5)
Overweight (25–< 30)	461	31.8 (1.5)	Missing	1	<0.3^c^
Obese (≥30)	478	34.6 (1.6)	Stomach illness^a^		
HDL <60 mg/dL			No	1165	85.2 (1.1)
No	477	30.7 (1.4)	Yes	115	9.0 (1.0)
Yes	895	69.3 (1.4)	Missing	92	5.8 (0.7)
Glucose ≥88 mg/dL			Physical health poor^a^		
No	698	46.6 (1.8)	0 days	874	64.5 (1.3)
Yes	674	53.4 (1.8)	1 to 3 days	178	13.2 (0.9)
CRP ≥ 1 mg/dL			4 to 6 days	78	5.8 (0.8)
No	1202	90.8 (0.7)	7 or more days	150	10.8 (1.0)
Yes	170	9.2 (0.7)	Missing	92	5.8 (0.7)
Anyone smoke in home?^a^			Mental health poor^a^		
No	1302	95.8 (0.7)	0 days	776	56.8 (1.8)
Yes	67	4.1 (0.7)	1 to 7 days	361	27.3 (1.4)
Missing	3	<0.3^c^	7 to 14 days	46	3.6 (0.7)
			15 to 30 days	97	6.4 (0.8)
			Missing	92	5.8 (0.7)

^a^past year; ^b^past 30 days; ^c^Weighted percentage less than 0.3 (SE not calculated); ^d^High blood pressure was determined using measured values at the examination; ^e^Self-reported that respondents were told by doctor or other health care provider that they had the condition; ^f^The weighted percent adjusts for differential probabilities of selection, nonresponse, and differences between the final sample and the total population

### Self-reported health (SRH)

**Fig. 1:**
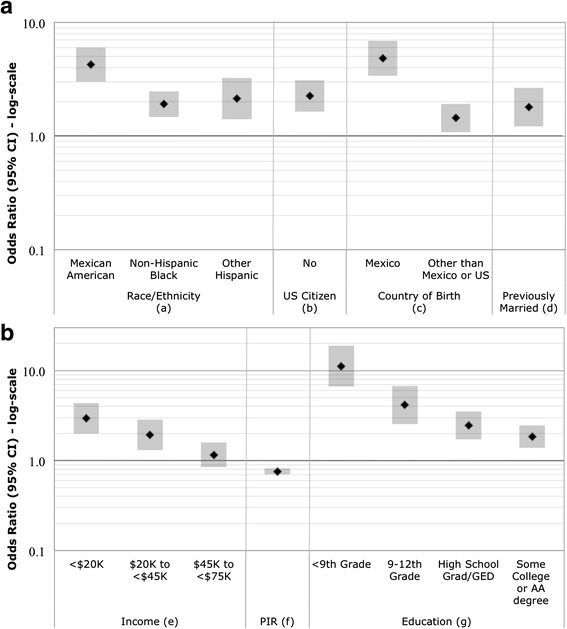
**a** and **b** Odds Ratios (95 % CI) of poorer SRH for sociodemographic variables. Odds Ratios are adjusted for age and sex. (*a*) Reference category is non-Hispanic White. (*b*) United States citizenship: index = no; referent = yes. (*c*) Reference category is born in the United States. (*d*) Marital status: index = widowed/divorced/separated; referent = married. (*e*) Reference category is an income of ≥ $75,000. (*f*) Family income to poverty ratio: continuous scale. (*g*) Reference category is an education level of ≥ college graduate

**Fig. 2:**
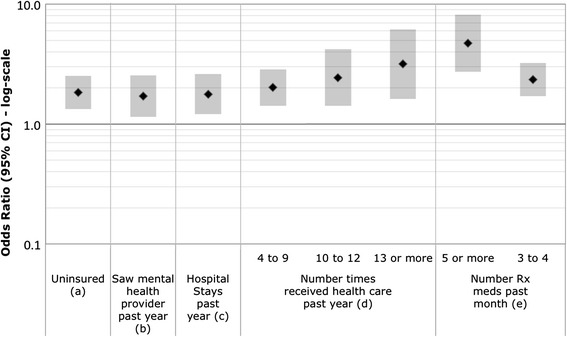
Odds Ratios (95 % CI) of poorer SRH for health care domain variables. Odds Ratios are adjusted for age and sex. (*a*) Health insurance: index = no; referent = yes. (*b*) Reference category is no. (*c*) Number of hospitals stays past year: index = at least 1; referent = none. (*d*) Reference category is 0 times. (*e*) Reference category is 0 prescription medications

**Fig. 3:**
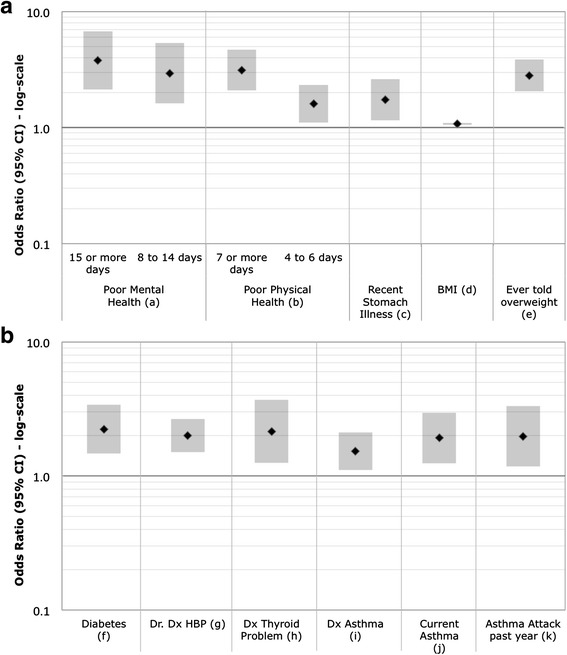
**a** and **b**. Odds Ratios (95 % CI) of poorer SRH for illnesses and mental health. Odds Ratios are adjusted for age and sex. (*a*) Number of days mental health was not good during the past 30 days: referent = 0 days. (*b*) Number of days physical health was not good during the past 30 days: referent = 0 days. (*c*) Had a stomach illness in the past 30 days: index = yes, referent = no. (*d*) Body mass index: continuous scale. (*e*) Ever told by a health care professional that you are overweight: index = yes; referent = no. (*f*) Diagnosed diabetes: index = yes and borderline; referent = no. (*g*) Doctor diagnosed high blood pressure: index = yes; referent = no. (*h*) Ever told by a health care professional that you have a thyroid problem: index = yes; referent = no. (*i*) Ever told by a health care professional that you have asthma: index = yes; referent = no. (*j*) Currently have asthma: index = yes; referent = no. (*k*) Had an asthma attack in the past year: index = yes, referent = no

**Fig. 4:**
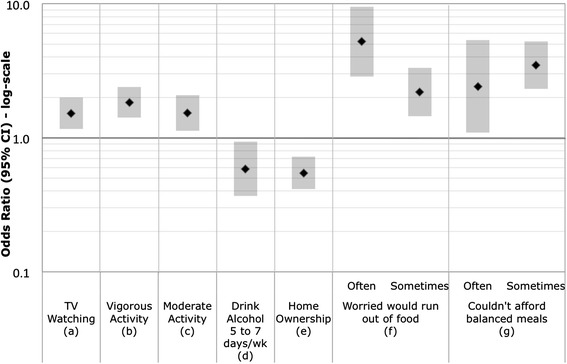
Odds Ratios (95 % CI) of poorer SRH for lifestyle domain variables. Odds Ratios are adjusted for age and sex. (*a*) Average hours per day watching television: index = 3 or more hours; referent = 2 or less hours. (*b*) Any vigorous activity in the last 30 days: index = no; referent = yes. (*c*) Any moderate activity in the last 30 days: index = no; referent = yes. (*d*) Days per week alcohol was consumed: referent = never drinks alcohol. (*e*) Home owned or rented: index = owned/being bought; referent = rented. (*f*) Reference category is never worried house would run out of food in the last 12 months. (*g*) Reference category is never true that could not afford balanced meals in the past 12 months

NHANES respondents were asked in a computer assisted personal interview: *would you say your health in general is excellent, very good, good, fair, or poor?* SRH was analyzed in two ways. First, SRH was collapsed into a binary variable that combined excellent, very good, and good into one category and fair and poor into a second category. This dichotomy is commonly used by others investigating SRH [24–27] and helps account for imbalances resulting from low numbers of respondents in the extreme lower ends of the scale (i.e., those reporting poor health). Second, SRH was considered a continuous ordinal measure (5 = Excellent to 1 = Poor) and modeled using ordinal logistic regression with the resultant odds ratios (OR) reflecting the odds of a respondent reporting poorer health. A comparison of the relationships between SRH categories and health risk indicators and ORs derived using the ordinal five-point response versus the binary responses are shown in (Additional file 1: Tables S1–S6). We present results from the ordinal SRH categories in the Figs. 1, 2, 3 and 4 and comparison for both the binary and ordinal responses in Tables 4 and 5.

### Race/ethnicity

Race/ethnicity was reported as a five-category variable in NHANES and derived from responses to survey questions on race and Hispanic origin. Respondents who self-identified as Hispanic of Mexican-American origin or ancestry were coded as “Mexican-American.” Respondents who self-identified as Hispanic of other Hispanic origins or ancestries (e.g., Puerto Rican, Cuban, and Dominican) were coded as “Other Hispanic.” Respondents who self-identified as non-Hispanic were then categorized based on their self-reported race: non-Hispanic White, non-Hispanic Black, and other non-Hispanic including multi-racial.

### Health risk indicators

Health risk factors were selected from NHANES based on their known ability to reflect or contribute to health status by direct or indirect pathways. There are over 1200 indicator variables to choose from, including over a hundred blood biomarkers of chemical exposure. Health risk indicator variables were extracted for 57 respondent characteristics across six domains.

**Domain 1** focused on sociodemographic factors: income and poverty-income ratio (PIR), high school education attainment, and marital status. **Domain 2** focused on health care factors: lack of health insurance, hospitalizations, number of times received health care, mental health visits, prescription medication use in the last 30 days, and Hepatitis A and B immunization. **Domain 3** focused on health status factors: mental and physical health, body mass index (BMI), high blood pressure, asthma, thyroid problems, diabetes, stomach illness, and cancer/malignancy. **Domain 4** focused on lifestyle behaviors: whether respondents were worried they would run out of food or could not afford balanced meals in the past year, hours spent watching TV, duration of moderate and vigorous monthly physical activity, alcohol consumption, and home characteristics. **Domain 5** focused on clinical indicators of poor health or pre-disease status: high-density lipoprotein (HDL), cholesterol (below 60), C-reactive protein (CRP; ≥1 μg/dL), serum glucose, glycohemoglobin (>7 %), and serological nutritional indicators of health including calcium, vitamin C, and vitamin D, cell counts and morphology, and blood iron markers. **Domain 6** focused on blood biomarkers of chemical exposure: cotinine, three metals (cadmium, lead, and mercury) and two volatile organic compounds (VOCs: benzene and toluene).

Additionally, we explored cumulative exposure to environmental chemicals. From the variables in Domain 6, we derived three environmental scores reflecting combinations of blood metal levels and VOCs. Environmental Score 1 combined blood lead and cadmium levels, Environmental Score 2 combined lead, cadmium, and mercury blood levels, and Environmental Score 3 combined benzene and toluene blood levels. These cumulative environmental scores were calculated by assigning participants a value of one if their blood chemical level was greater than the median blood level in the population studied and a value of zero for each blood chemical level less than or equal to the median blood level. For example, Environmental Score 1 had a range of 0 to 2.

### Statistical analysis

The outcome of interest for the study was poorer SRH. The predictors of interest were the variables within the six domains and environmental scores described above. ORs and 95 % CIs were calculated using binary and ordinal logistic regression. All analyses were carried out with SAS 9.3 (SAS Institute, Inc., Cary, North Carolina) and incorporated the appropriate sample weights to adjust for differential probabilities of selection, nonresponse, and differences between the final sample and the total population. The NHANES stratification and clustering design variables were used in the binary and ordinal logistic regression modeling to obtain proper standard errors of the estimates. Models were adjusted for age and sex for all domains. We derived an indicator variable for three clinical indicators with widely recognized cut-offs for assessing health: CRP, HDL, and vitamin D. A subject’s continuous data points were transformed and assigned a value of 1 or 0 for these three derived variables with a value of 1 representing an indicator of a poorer health quality (CRP ≥1 mg/dl, HDL < 60 mg/dL, and vitamin D <20 ng/mL). When predicting poorer SRH for the serological health risk indicators, the models were additionally adjusted for asthma and diabetes, two diseases known to significantly impact SRH. For the serological nutritional and health risk indicators, the median levels or physiological relevant cut-points were applied to the whole NHANES population of 20 to 50 year olds that had data for the indicator of interest. For the blood biomarkers of exposure, the median levels were calculated based on the 20 to 50 year old-nonsmokers (with less than 10 ng/ml of cotinine) who had data for the chemical of interest. To calculate the median values for environmental exposures, individuals with values below the limit of detection (LOD) were assigned the LOD divided by the square root of two, a methodology used by the National Center for Health Statistics at Centers for Disease Control and Prevention.

## Results

The full set of characteristics and health indicators can be found in Tables 1 and 2, which display the frequency and weighted percent of the characteristics of the study participants and the indicators. Additional file 1: Tables S1–S6 includes all health risk factors examined along with associations of poorer reports of health.

Table 1 presents descriptive demographic statistics of this study sample. The mean age of study respondents was 36. Among these, 54 % were women, and 9 % reported poor/fair SRH (*N* = 187). Approximately 70 % of respondents had some college education or higher. Sixty percent had annual incomes greater than $45,000, while 14 % had annual incomes less than $20,000. The majority of respondents were non-Hispanic White (68 %), while 11 % were non-Hispanic Black, 11 % Mexican American, 5 % other Hispanic, and 5 % other race including multi-racial.

Table 2 highlights selected lifestyle, health care, health status, dietary and clinical indicators, and environmental exposure characteristics. The majority of the respondents did not have self-reported current asthma (92 %), self-reported diabetes (96 %), self-reported thyroid problems (93 %), or high blood pressure (89 %) as measured at the examination. Table 3 provides the distributions of the continuous variables used in the analysis.

**Table 3:** Distribution of continuous variables of interest in the study population (20–50 year old nonsmokers)

	Arithmetic mean (SE)	Geometric mean (95 % CI)	Median	IQR	5^th^, 95^th^ percentile	LOD	% below LOD
Participant characteristics							
Age (years)	36.4 (0.32)		36.5	14.4	21.2, 48.8		
BMI	28.4 (0.22)		27.7	8.1	19.8, 39.1		
Systolic BP (mm Hg)	117.9 (0.54)		115.5	16.9	96.9, 140.8		
Diastolic BP (mm Hg)	72.0 (0.48)		70.9	15.2	52.8, 91.5		
PIR	3.3 (0.08)		3.4	3.1	0.6, 5.0		
Clinical indicators							
Vitamin D (ng/mL)	23.8 (0.45)	21.9 (21.0, 23.0)	22.7	11	8.90, 38.5	5	0.21
Vitamin C (mg/dL)	0.98 (0.02)	0.85 (0.81, 0.89)	0.98	0.56	0.24, 1.70	0.012	0
Total calcium (mg/dL)	9.50 (0.02)	9.49 (9.46, 9.53)	9.45	0.43	8.93, 9.96	2	0
Glucose, Serum (mg/dL)	92.4 (0.67)	90.5 (89.4, 91.5)	88.1	13.1	73.6, 117.0	3	0
Direct HDL-Cholesterol (mg/dL)	54.1 (0.54)	52.1 (51.1, 53.1)	51.3	20.5	33.5, 80.9	^a^	
CRP (mg/dL)	0.43 (0.04)	0.17 (0.15, 0.18)	0.15	0.34	0.02, 1.41	0.02	2.8
Iron markers							
Transferrin receptor (mg/L)	3.81 (0.06)	3.60 (3.51, 3.68)	3.4	1.4	2.11, 6.36		
Ferritin (ng/mL)	56.98 (2.77)	37.14 (34.96, 39.46)	39.2	45	5.65, 177.5		
Hematocrit (%)	42.4 (0.16)	42.2 (41.9, 42.5)	42.4	6.3	35.5, 49.4		
Hemoglobin (g/dL)	14.4 (0.05)	14.3 (14.2, 14.4)	14.4	2	11.8, 16.8		
Mean cell hemoglobin (pg)	30.2 (0.10)	30.1 (29.9, 30.3)	30.4	2.3	26.6, 32.7		
MCHC (g/dL)	33.9 (0.07)	33.9 (33.8, 34.0)	33.8	1	32.4, 35.1		
Protoporphyrin (μg/dL RBC)	64.6 (1.51)	58.2 (56.6, 59.9)	53.2	26	34.0, 126.6		
Iron, Frozen serum (μg/dL)	76.8 (1.47)	68.8 (66.5, 71.2)	71.5	46	28.5, 134.2		
TIBC, Frozen serum (μg/dL)	365.1 (2.94)	359.9 (355.3, 364.6)	359	80	277.1, 481.2		
Transferrin saturation (%)	21.8 (0.51)	19.1 (18.4, 19.9)	19.9	14.1	7.00, 39.2		
Environmental exposure							
Blood lead (μg/dL)	1.34 (0.04)	1.09 (1.04, 1.15)	1.06	0.88	0.46, 2.96	0.3, 0.25	0.4
Blood cadmium (μg/L)	0.27 (0.006)	0.23 (0.22, 0.23)	0.21	0.18	0.10, 0.59	0.14, 0.2	34.7
Blood mercury, total (μg/L)	1.73 (0.09)	1.08 (0.97, 1.19)	1.02	1.48	0.21, 5.58	0.2, 0.33	7.6
Blood benzene (ng/mL)	0.030 (0.002)	0.024 (0.22, 0.026)	0.017	0.014	0.017, 0.060	0.024	57.2
Blood toluene (ng/mL)	0.162 (0.034)	0.087 (0.078, 0.097)	0.084	0.075	0.024, 0.308	0.025	4.4
Serum cotinine (ng/ml)	0.27 (0.04)	0.053 (0.046, 0.062)	0.04	0.1	0.01, 1.24	0.015	21.7

*Abbreviations: IQR* interquartile range, *LOD* limit of detection, *BMI* body mass index, *PIR* ratio of family income to poverty, *CRP* C-reactive protein, *MCHC* mean corpuscular hemoglobin, *TIBC* total iron binding capacity

^a^The detection limit for HDL is not found in the NHANES laboratory method for HDL

Figures 1, 2, 3 and 4 show ORs for poorer SRH health derived from ordinal logistic regression in association with race/ethnicity and the 57 health-risk indicators. All associations are adjusted for age and gender. Additional file 1: Tables S1–S6 compares the ORs for the ordinal five-point and binary (poor/fair versus good very/good/excellent) responses in association with race/ethnicity and all 57 health-risk indicator variables.

### Domain 1: Sociodemographic status

Figure 1 shows associations with SRH and sociodemographic characteristics. Mexican Americans, non-Hispanic Blacks, and other Hispanics reported poorer SRH than non-Hispanic Whites (Fig. 1A). Participants who were not U.S. citizens, were born outside of the U.S., or were widowed/divorced/separated also had poorer SRH. Lower income and education levels were consistently associated with poorer SRH. The PIR, which is an index for the ratio of family income to poverty, was associated with poorer SRH. Marital status, defined as living with a partner or never married versus married, was not associated with poor SRH (see Additional file 1: Table S1 “Sociodemographic Domain”).

### Domain 2: Health care

Figure 2 shows associations between indicator variables of health care access and utilization and reports of poorer SRH. Lack of health insurance, number of times the participant received health care over the past year, and taking prescribed meds over the past month were associated with poorer SHR (ordinal five point and binary SRH responses). In contrast, whether a participant had seen a mental health professional over the past year, was associated with poorer SRH only for the ordinal five point SRH responses. Receiving fewer doses (<2 versus at least 2) Hepatitis A vaccine immunizations was associated with a better SRH for the binary but not ordinal five point SRH responses. Hepatitis B vaccination was not associated with poorer SRH for either the binary or ordinal five-point responses (see Additional file 1: Table S2 “Health Care Domain”).

### Domain 3: Health status

Figure 3 presents associations between SRH and indicators of mental and physical health. Having more than 8 days in the past 30 when mental health was not good was associated with poorer SRH, as were the number of days physical health was not good during the past 30 days, BMI, diabetes, doctor-diagnosed high blood pressure, ever being told you have a thyroid problem, diabetes, ever told you were overweight, or had an asthma diagnosis, asthma attack last year, and having stomach illness.

Generally, the relationship between these health status indicators was consistent with the binary and the ordinal five-point responses for SRH, except asthma and stomach illness, which were not significant in the binary model. Ever being told you have cancer/malignancy was not associated with SRH by either the binary or ordinal five-point responses (see Additional file 1: Table S3 “Health Status Domain”).

### Domain 4: Lifestyle

Figure 4 presents associations between SRH and lifestyle factors. Respondents were more likely to report poorer SRH if they also reported being worried that the household would run out of food in the last 12 months (often and sometimes) or they reported not being able to afford balanced meals in the past 12 months. Watching more than three hours of TV daily or having no vigorous or moderate activity in the last 30 days were all associated with poorer SHR. Living in an apartment versus a single family home, or living in a mobile home or trailer versus detached single family home, were associated with poorer SRH, while owning versus renting a home and consuming alcohol (5–7 days/week vs. never) were less likely to report poorer SRH. No relationships were observed between SRH and number of persons in the household, number of rooms in the home, year home was built, and home type (attached single family house [SFH] vs. something else and something else vs. detached SFH) (see Additional file 1: Table S4 “Lifestyle Domain”).

### Domain 5: Serological clinical indicators

Table 4 shows the age, sex, asthma, and diabetes adjusted ORs for poorer SRH for the 35 clinical measures of nutrition, health risk, cell counts and morphology, and markers of iron status. In total, twenty-two of the thirty-five clinical health indicators (62 %) were associated with poorer SRH. Of the 11 included markers of blood iron status, nine (88 %) were associated with poorer SRH: hemoglobin, mean cell hemoglobin, mean corpuscular cell hemoglobin, red blood cell distribution width (RDW), transferrin receptor, transferrin saturation, glycohemoglobin, protoporphyrin, and serum iron. Of the 14 factors related to blood cells and morphology, (five) 36 % were associated with poorer SRH: platelet count, mean platelet volume, eosinophil and lymphocyte numbers, and mean cell volume. White cell count, basophils (number and percent), monocytes (number and percent), segmented neutrophils (number and percent), lymphocytes (%), and mean platelet volume were health factors not associated with poorer SRH. A comparison of the relationships between SRH categories and clinical risk indicators and ORs derived using the ordinal five-point response verses the binary responses is shown in Table 4.

**Table 4:** Odds Ratios^a^ of poorer SRH for the clinical domain

	*N*	Poorer SRH^b^		Fair/Poor SRH^c^	
		OR (95 % CI)		OR (95 % CI)	
Nutrition markers:					
Vitamin C (ng/mL)	1372	0.59 (0.43,0.82)	**	0.41 (0.25,0.67)	***
Vitamin D (ng/mL)	1372	0.96 (0.95,0.97)	****	0.94 (0.92,0.95)	****
Calcium (mg/dL)	1372	0.46 (0.34,0.63)	****	0.33 (0.18,0.61)	***
Disease risk factors:					
Serum Glucose > median (mg/dL)	1372	1.14 (0.92,1.40)	NS	1.01 (1.00,1.02)	**
CRP ≥ 1.0 mg/dL	1372	1.42 (1.00,2.03)	NS	2.02 (1.39,2.95)	***
Total Cholesterol (mg/dL)	1372	1.00 (1.00,1.00)	NS	1.00 (0.99,1.00)	NS
Low HDL, Yes vs. No	1372	1.92 (1.51,2.45)	****	1.68 (1.01,2.79)	*
Direct HDL-Cholesterol (mg/dL)	1372	0.98 (0.97,0.98)	****	0.98 (0.97,0.99)	**
Triglycerides	1372	1.00 (1.00,1.00)	**	1.00 (1.00,1.00)	NS
Cell counts & morphology:					
WBC count (1000 cells/μL)	1370	1.06 (0.99,1.13)	NS	1.07 (0.99,1.16)	NS
Platelet count SI (1000 cells/μL)	1370	1.00 (1.00,1.00)	*	1.00 (1.00,1.00)	NS
Basophils number	1360	1.03 (0.09,11.52)	NS	0.28 (0.01,9.98)	NS
Basophils percent (%)	1360	0.90 (0.69,1.18)	NS	0.89 (0.54,1.47)	NS
Monocytes number	1360	1.15 (0.53,2.49)	NS	1.38 (0.57,3.33)	NS
Monocytes percent (%)	1360	0.96 (0.91,1.01)	NS	0.99 (0.90,1.09)	NS
Segmented neutrophils number	1360	1.05 (0.96,1.14)	NS	1.08 (0.99,1.19)	NS
Segmented neutrophils percent (%)	1360	1.00 (0.98,1.02)	NS	1.01 (0.99,1.03)	NS
Eosinophils number	1360	2.03 (1.13,3.64)	*	3.62 (0.97,13.52)	NS
Eosinophils percent (%)	1360	1.02 (0.97,1.07)	NS	1.06 (0.95,1.19)	NS
Lymphocytes number	1360	1.23 (1.02,1.48)	*	0.99 (0.71,1.37)	NS
Lymphocytes percent (%)	1360	1.00 (0.99,1.02)	NS	0.98 (0.96,1.01)	NS
Mean platelet volume (fL^d^)	1370	1.01 (0.87,1.18)	NS	1.27 (1.00,1.61)	*
Mean cell volume (fL^d^)	1372	0.96 (0.93,0.99)	**	0.93 (0.91,0.96)	****
Iron markers:					
Hematocrit (%)	1370	0.97 (0.93,1.01)	NS	0.95 (0.88,1.03)	NS
Hemoglobin (g/dL)	1370	0.89 (0.80,0.99)	*	0.81 (0.65,1.01)	NS
MCHC (g/dL)	1370	0.89 (0.80,1.00)	*	0.66 (0.50,0.86)	**
Mean cell hemoglobin (pg)	1370	0.91 (0.86,0.96)	***	0.83 (0.77,0.89)	****
Red cell distribution width (%)	1370	1.26 (1.13,1.40)	****	1.34 (1.14,1.58)	***
Ferritin (ng/mL)	801	1.00 (1.00,1.00)	NS	1.00 (0.99,1.00)	NS
Transferrin receptor (mg/L)	792	1.14 (1.04,1.25)	**	1.26 (1.11,1.43)	***
Transferrin saturation (%)	825	0.98 (0.97,1.00)	**	0.94 (0.91,0.96)	****
Glycohemoglobin (%)	1368	1.63 (1.36,1.96)	****	1.82 (1.30,2.55)	***
Protoporphyrin (μg/dL RBC)	1372	1.01 (1.00,1.01)	****	1.01 (1.00,1.01)	***
Iron, Frozen Serum (μg/dL)	825	0.99 (0.99,1.00)	**	0.98 (0.97,0.99)	****
TIBC, Frozen Serum (μg/dL)	825	1.00 (1.00,1.00)	NS	1.00 (1.00,1.01)	NS

*NS* not significant; *p < 0.05; **p < 0.01; ***p < 0.001; ****p < 0.0001

^a^Odds Ratios adjusted for age, sex, diagnosed asthma, and diabetes

^b^OR estimated with ordinal logistic regression of 5 point response of excellent to poor self-reported health

^c^OR estimated with logistic regression on a binary SRH outcome that collapses excellent, very good, and good into one category and fair and poor into a second category

^d^fL = femtoliters

### Domain 6: Blood biomarkers of chemical exposure

Table 5 shows the age and sex adjusted ORs for respondents reporting poorer SRH for biomarkers of chemical exposures. Blood cadmium and lead levels were associated with poor/fair SRH, while blood mercury levels were associated with better SRH (good/very good/excellent health). Environmental Score 1 considered the cumulative effect of blood lead plus cadmium indicated that if both lead and cadmium blood levels were greater than the median (as compared to only one of the blood metals), the odds of poorer SRH increased from an OR of 1.87 (95 % CI: 1.18, 2.96) to 3.47 (95 % CI: 2.26, 5.34). Environmental Score 2, which considered the cumulative effect of lead plus cadmium and mercury blood levels, was associated with a poorer SRH as compared to when either one or two of the metals was greater than the median: 2.25 (95 % CI: 2.38, 3.96) as compared to 1.98 (95 % CI: 1.06, 3.68). To verify that the blood cadmium and lead levels were not confounded by passive exposure to cigarette smoke, we adjusted for cotinine; however, this further adjustment did not impact the associations. Environmental Score 3, which considered the cumulative effect of benzene and toluene, was not associated with SRH. The differences between the ordered logit and binary logit models, in terms of statistical significance, was particularly apparent for the Environmental scores 1 and 2. These discrepant results tend to be those with the smallest number of respondents. For example, in Environmental Score 1, respondents in this category were as low as 374 respondents. This could affect the accuracy of the assumption that the odds ratio is constant across categories of self-reported health, especially since there are very few reporting “Poor” health.

**Table 5:** Odds Ratios^a^ of poorer SRH for the environmental biomarker exposure domain

	*N*	Poorer SRH^b^		Fair/Poor SRH^c^	
		OR (95 % CI)		OR (95 % CI)	
Does anyone smoke in the home? (y vs. n)	1369	1.54 (0.92,2.59)	NS	1.40 (0.65,3.02)	NS
Cotinine (ng/mL)	1372	1.12 (1.00,1.25)	NS	1.04 (0.90,1.20)	NS
Cadmium (μg/L)	1372	1.76 (1.14,2.74)	*	2.65 (1.36,5.14)	**
Lead (μg/dL)	1372	1.14 (0.99,1.30)	NS	1.28 (1.03,1.58)	*
Mercury, total (μg/L)	1372	0.91 (0.86,0.95)	****	0.75 (0.64,0.88)	***
Toluene (ng/mL)	1372	1.06 (1.03,1.10)	***	0.99 (0.89,1.11)	NS
Benzene (ng/mL)	1372	0.41 (0.15,1.16)	NS	0.59 (0.06,5.75)	NS
Toluene (below DL vs. above DL)	1372	1.61 (0.76,3.41)	NS	1.21 (0.53,2.77)	NS
Benzene (below DL vs. above DL)	1372	1.11 (0.87,1.40)	NS	0.92 (0.64,1.32)	NS
Environmental Score 1^d^	1372				
Pb & Cd > median vs. neither	374	1.37 (0.98,1.93)	NS	3.47 (2.26,5.34)	****
Pb or Cd > median vs. neither	581	1.01 (0.76,1.34)	NS	1.87 (1.18,2.96)	**
Environmental Score 2^e^	1372				
Pb, Cd, & Hg > median vs. none	208	1.17 (0.80,1.71)	NS	2.25 (1.28,3.96)	**
2 of Pb, Cd, or Hg > median vs. none	475	1.29 (0.81,2.04)	NS	1.98 (1.06,3.68)	*
Pb, Cd, or Hg > median vs. none	445	1.20 (0.78,1.84)	NS	1.68 (0.88,3.20)	NS
Environmental Score 3^f^	1372				
Benzene & Toluene > median vs. neither	400	1.22 (0.92,1.63)	NS	1.21 (0.75,1.94)	NS
Benzene or Toluene > median vs. neither	452	1.20 (0.88,1.65)	NS	1.05 (0.60,1.84)	NS

*NS* not significant; *p < 0.05; **p < 0.01; ***p < 0.001; ****p < 0.0001

^a^Odds Ratios adjusted for age and sex

^b^OR estimated with ordinal logistic regression of the 5 point response scale of excellent to poor self-reported health

^c^OR estimated with logistic regression on a binary SRH outcome that collapses excellent, very good, and good into one category and fair and poor into a second category

^d^Pb and Cd (1 if > median of nonsmokers)

^e^Pb, Cd, and Hg (1 if > median of nonsmokers)

^f^Benzene and toluene (1 if > median of nonsmokers)

## Discussion

Effective planning and decision-making for improving the health of a community requires information about the current health status and individual factors that will influence health status [28]. SRH was used to delineate and explore relationships between race/ethnicity and 56 potentially modifiable population health determinants across six domains: sociodemographic, health care, health status (e.g., diseases/health conditions), lifestyle factors, serological clinical and nutritional indicators, and blood biomarkers of exposures for metals and volatile organic compounds. Individual-level data was combined from two NHANES reports (2003–2006) for 1372 nonsmoking adults.

Poorer SRH was associated with race/ethnicity, citizenship, income and education level, lack of health insurance, number of hospitalizations, food security, exercise, poor mental and physical health, prescription drug use, health outcome measures (e.g., diabetes, thyroid problems, asthma, stomach illness), several serological levels of nutrition, clinical measures of health risk, and blood biomarkers of environmental exposures for lead, mercury, cadmium, and toluene, but not benzene.

We note general consistency between the ordered logit and binary logit models in terms of statistical significance, but there are a few differences of note. The discrepancies may be explained in part by the different assumptions of the two models. The ordered logit model assumes the effect is constant for each category of self-reported health (i.e., the effect of poorer heath from Excellent to Good is the same as Fair to Poor); whereas, the binary model assumes that those reporting Fair and Poor and those reporting Excellent, Very Good, and Good categories are similar enough to be grouped together. While some information is certainly lost by this grouping, as we noted previously, this is a fairly common practice in studies of SRH [24–27] and overcomes issues related to low numbers in some of the categories, especially the “Poor” response which was only by 21 (1.2 %) of respondents in our sample (Table 2). The binary model also does not require an assumption related to constancy of the odds ratio across multiple categories. These two models provide complementary but different interpretations of the association between SRH and the health risk indicators. For a more rigorous comparison and discussion of binary and several ordered SRH analytic choices, see Manor et al. [29] and Barger [30].

Mexican Americans and non-Hispanic Blacks, when compared to non-Hispanic Whites, were more likely to report poorer SRH. These findings are consistent with Shetterly et al. [5] and Benjamins et al. [31]. Our analyses showed a strong association with poorer SRH with lower education and income levels. Lahelma et al. [32] explain the clear associations between health and education, occupational class, and family income. Adler and Ostrove [33] discussed how sociodemographic and environmental factors, individual psychological and behavioral factors, and biological predispositions and processes can impact health status.

Associations were observed between poorer SRH and the number of days a respondent’s mental health was not good. This finding suggests that SRH incorporates a mental health or psychosocial component that otherwise would go undetected in serological based clinical tests.

Poorer SRH was associated with lower levels of Vitamin C, Vitamin D, and calcium. These findings are consistent with Radimer et al. [34], who showed that intake of multivitamin and multi-minerals dietary supplements by US adults was associated with very good/excellent self-reported health. Poorer SRH was associated with lower levels of HDL, higher levels CRP, triglycerides, serum glucose, glycohemoglobin, platelet count, elevated eosinophil, and lymphocyte number. Nine of eleven blood iron markers were associated with SRH. These health indicators are linked to cardiac health, diabetes risk, and other medical conditions. Of particular note in our study was the strong association between the RDW and poorer SRH. Several studies have reported strong associations between RDW and mortality, although the mechanism by which RDW influences health status is unknown [35–37]. Based on the strong associations observed between RDW and SRH and because RDW is routinely performed, RDW may serve as an important early indicator of adverse health status prior to disease onset. 

### Biomarkers of chemical exposure

Blood levels of the three toxic heavy metals (cadmium, lead, mercury) and two VOCs (toluene and benzene) were evaluated in relation to SRH. All five chemicals have public health importance due to their environmental abundance and well-documented toxicity. SRH was associated with blood levels of cadmium, mercury, lead, and toluene but not benzene (perhaps because only 43 % of the respondents in this study were above the limit of detection for benzene). Examination of benzene in relation to health is of interest in light of studies showing ambient air levels of benzene and formaldehyde contribute nearly 60 % of the total cancer-related health impacts of air pollution in the United States [38].

People are exposed to mixtures of pollutants, through a variety of media, including air, water, and food. Thus, research is needed to better understand the cumulative risks posed to human health from the myriad of environmental contaminants that can occur simultaneously. Interactive effects of chemical within mixtures are complex and can result in alterations in the distribution, metabolism, absorption, and excretion of the chemicals [39]. Recently, Cobbina et al. [40] observed synergistic effects of metals mixtures which is consistent with our data where the odds of reporting poorer SRH were greater if the combined blood levels of mercury, lead, and cadmium were considered as opposed to each of the individual metals. In isolation, increasing levels of blood mercury were associated with a better SRH, an association that is likely confounded by income and fish consumption. For example, Mahaffey et al. [41] showed that blood mercury levels in women was related to higher income, consumption of fish, ethnicity, and residence (census region and coastal proximity). Higher blood lead and cadmium levels were associated with lower income levels [42]. Taken together, these studies underscore the need for further research into the relationships between health and cumulative exposures to chemicals, in the context of cultural, economic factors, especially for vulnerable populations and communities [43].

Our data suggest that SRH may be a useful screening-level indicator of health status for community-based health and environmental studies based on the number of associations of SRH of several sociodemographic, health care, health, lifestyle, serum-based nutritional, and serum-based environmental measurements. Examples of studies using screening level indices are those by Gallagher et al. [44] where health, sustainability, and environmental indices were derived for fifty major US cities. These diverse indices and associated indicators from which they were derived were associated with disparities related to race, education, and income. Messer et al. [45] applied a multidimensional neighborhood deprivation index (which considered income/poverty, education, employment, housing, and occupation) in relation to adverse prenatal events. Major et al. [46] applied the same index to evaluate associations with all-cause cancer, cardiovascular disease, and mortality. Derivation of an environmental quality index holds promise for improving the linkage between the impact of the overall environment and health [47].

### Limitations

As a single-question qualitative measurement, SRH is unable to capture all aspects of health risk or health status. Burgard and Chen [48] suggested that the comparability of self-reported information about specific health conditions might vary across race and social groups, in part because of diagnosis bias. Additionally, measures of specific symptoms may differ if respondents interpret questions or concepts differently. In this analysis, the study population was limited to 20–50 year old nonsmokers, which limits the generalizability of our findings for children and the elderly. We selected this age range in part because some of the blood chemical concentrations were only available for 20–50 year olds. Additionally, the elderly have higher rates of morbidity and children are undergoing rapid developmental changes that may lead to more varied clinical and nutritional measures. Due to the cross-sectional design of the study, we cannot infer causality as the basis for any relationships observed between the explanatory variables and SRH. We did not conduct analyses to evaluate possible correlations between and amongst variables within each of the domains. Further, it is likely that many of the social factors that affect health have both independent and interactive effects on various measures of health. For example, low income is often associated with many other factors contributing to poor health outcomes (e.g., lower levels of education, substandard housing, risky health behaviors, food insecurity, and lack of health insurance coverage). Because this was an exploratory, hypothesis generating analysis, multiple testing correction approaches were not applied. Therefore, *p*-values should be interpreted with caution. In addition, multivariate regression models were not evaluated.

## Conclusion

SRH was used to delineate and explore relationships between multiple health risk factors that ultimately will help inform the design of subsequent studies by highlighting risk factors that relate to health status. To the best of our knowledge, no previous research has applied both binary and ordered logit models to study the relationships between SRH for such a wide range of health risk factors. Nonsmoking respondents representative of the United States population reported poorer SRH in associations with race/ethnicity, income and education level, and a majority of the health risk indicators studied, including serological measures of nutrition and health risk and blood biomarkers of environmental exposures.

Our analyses, along with others [3, 12, 17, 49–51], lend support for the utility and continued validation of SRH as a reasonable proxy of health status for application in screening level community-based health and environmental studies, to identify vulnerable neighborhoods or counties, guide and prioritize public policy decisions in communities with suspected health disparities, and assist with exposure assessments, which often lack individualized health data.

## Abbreviations

BMI, body mass index; Cd, cadmium; CI, 95 % confidence intervals; CRP, C-reactive protein; HDL, high-density lipoprotein; Hg, mercury; LOD, limit of detection; NHANES, National Health and Nutrition Examination Survey; ORs, odds ratios; Pb, lead; PIR, ratio of family income to poverty; RDW, red blood cell distribution width; SFH, single-family house; SRH, self-reported health; VOCs, volatile organic compounds

## Additional file

Additional file 1:
**Table S1.** Sociodemographic Domain; **Table S2.** Health Care Domain; **Table S3.** Health Status Domain; **Table S4.** Lifestyle Domain; **Table S5.** Clinical Indicators; **Table S6.** Environmental Scores/Chemicals). This file details all associations between the domain factors and poorer SRH for both binary (poor/fair versus good/very good/excellent) and ordinal 5-point scoring of SRH (1 = poor, 2 = fair, 3 = good, 4 = very good, and 5 = excellent). (XLSX 45 kb)
